# Early Risk stratification for Arrhythmic death in Patients with ST-Elevation Myocardial Infarction

**Published:** 2007-01-01

**Authors:** Majid Haghjoo, Reza Kiani, Amir Farjam Fazelifar, Abolfath Alizadeh, Zahra Emkanjoo, Mohammad Ali Sadr-Ameli

**Affiliations:** Department of Pacemaker and Electrophysiology, Rajaie Cardiovascular Medical and Research Center, School of Medicine, Iran University of Medical Sciences, Tehran, Iran

**Keywords:** myocardial infarction, sudden death, risk stratification, heart rate variability, signal-averaged electrocardiography, ejection fraction, non-sustained ventricular tachycardia

## Abstract

**Background:**

Sudden cardiac death is a leading cause of death in patients with ST-elevation myocardial infarction (MI). According to high cost of modern therapeutic modalities it is of paramount importance to define protocols for risk stratification of post-MI patients before considering expensive devices such as implantable cardioverter-defibrillator.

**Methods:**

One hundred and thirty seven patients with acute ST-elevation MI were selected and underwent echocardiographic study, holter monitoring and signal-averaged electrocardiography (SAECG). Then, the patients were followed for 12 ±3 months.

**Results:**

During follow-up, 13 deaths (9.5%) occurred; nine cases happened as sudden cardiac death (6.6%). The effect of ejection fraction (EF) less than 40% on occurrence of arrhythmic events was significant (P<0.001). Sensitivity and positive predictive value of EF<40% was 100% and 76.95% respectively. Although with lesser sensitivity and predictive power than EF<40%, abnormal heart rate variability (HRV) and SAECG had also significant effects on occurrence of sudden death (P=0.02 and P=0.003 respectively). Nonsustained ventricular tachycardia was not significantly related to risk of sudden death in this study (P=0.20).

**Conclusion:**

This study indicated that EF less than 40% is the most powerful predictor of sudden cardiac death in post MI patients. Abnormal HRV and SAECG are also important predictors and can be added to EF for better risk stratification.

## Introduction

Arrhythmic cardiac events are a common cause of death in patients with ST-elevation myocardial infarction (MI) and perhaps the most preventable cause. Due to large number of these patients and high cost of implantable cardioverter-defibrillator (ICD) implantation, selection of eligible patients is very important. Factors such as low ejection fraction (EF), abnormal signal-averaged electrocardiogram (SAECG), abnormal heart rate variability (HRV), nonsustained ventricular tachycardia (NSVT) and T-wave alternans (TWA) have been used for risk stratification of postMI patients [1-3]. Among them EF seems to be more important.

However it is not practical to select patients based on low EF alone because too many patients would be ICD candidates [4]. On the other hand other methods such as SAECG and HRV seem to have additive value to low EF when they are used together [5,6]. The purpose of this study was to determine value of low EF, abnormal SAECG, HRV, and NSVT as the risk factors of post MI sudden cardiac death as well as the effect of revascularization on reducing the risk of arrhythmic death in these patients.

## Methods

### Patient characteristics

Included were 137 patients admitted with diagnosis of acute ST-elevation MI in coronary care units at our institution. The study was approved by the local Ethics Committee, and written informed consents were obtained from all the included patients. None of the patients included in this study had standard class I indication for ICD implantation.

Acute ST-elevation MI requires all of the following three criteria: (1) *Chest pain*: more than 30 minutes or equivalent symptoms; (2) *ECG*: ≥0.1 mV ST-elevation in two adjacent limb leads, and/or ≥0.2 mV ST elevation in two adjacent precordial leads, or left bundle branch block, or appearance of new Q-waves (≥0.03 s); (3) *Enzymes*: CK-elevation (≥ twice the upper normal limit) and CK-MB >24 units/l, or troponin positive. All patients underwent echocardiographic study, 24-hr ambulatory electrocardiography and SAECG at fifth or sixth day of admission. Mean age at time of study was 59.5±11.3 years (range 32-90 years), and 76.6% of the patients were males. Seventy-one patients (51.8%) experienced anterior MI and 66 patients (48.2%) had inferior/posterior MI. The patients were followed for 12±3 months. After completion of follow-up period, information about life status, possible arrhythmias and probable revascularization extracted from their medical records or via telephone call or interview.

### Holter recording

Each patient underwent 24-hour Holter recording (VISTA®, Novacor, France). During Holter recording, patients were asked to note the timing if any symptom occurred. To check for NSVT, we used a full-disclosure method that allowed review of all 24-hour of ECG recording. A cardiologist, blinded to presenting symptom read the reports and ECG printouts of arrhythmias during symptomatic and asymptomatic episodes. NSVT was defined as occurrence of three or more consecutive premature ventricular beat with rate of ≥100 bpm and abnormal HRV as SDNN ≤ 70 ms.

### Signal-averaged electrocardiography

The SAECG was recorded using a commercially available system (Hellige EK 56; Marquette Hellige, Freiburg, Germany). Before SAECG recording, the skin was prepared by shaving, removing skin debris with alcohol, and abrading the skin with gauze. The SAECG was recorded with standard bipolar X, Y, and Z orthogonal leads. Signals were amplified, averaged, and filtered with a bidirectional filter at frequencies of 40-250 Hz. About 200 beats were averaged to a noise level of <0.5 μV before signal amplification and filtering. The filtered signals were combined into a vector magnitude V and the QRS duration, the duration of low amplitude signals < 40 μV (LAS 40), and the root mean square voltage of the signals in the last 40 ms of the filtered QRS (RMS 40) were calculated.

The SAECG was considered to be abnormal if two of the following three criteria were met: (1) total filtered QRS duration > 114 ms; (2) RMS 40 < 20 μV; and (3) LAS 40 > 38 ms. Otherwise, the SAECGs were classified as normal [7].

### Echocardiography

Standard echocardiography with Doppler studies was performed (Vivid 7, General Electric, USA). Left ventricular (LV) end-systolic and diastolic dimensions and volumes and LVEF were measured by two-dimensional guided M-mode echocardiography according to the guidelines of the American Society of Echocardiography [8].

### Statistical analysis

Continuous data are presented as mean±SD and ranged when appropriate. Continuous variables were compared by Student's t-test in case of normal distribution. Otherwise, nonparametric test of Mann-Whitney U was used. Chi-square analysis was used for categorical data and Fisher exact test for cell count less than five. Sensitivity, specificity, positive predictive value, and negative predictive value of EF, SAECG, HRV, and nonsustained VT were measured according to standard formula and ROC curve analysis. A two-tailed P-value < 0.05 was defined statistically significant. The software SPSS version 13.0 (SPSS Inc., Chicago, Illinois) was used for statistical analysis.

## Results

Mean age of patients at time of study was 59.5±11.3 years, and 76.6% of the patients were males. Seventy-one patients (51.8%) experienced anterior MI and 66 patients (48.2%) had inferior/posterior MI (Table 1). The patients were followed for 12±3 months. There were 13 deaths in the follow-up period. Nine of these deaths (69%) occurred suddenly. Of these 9 patients, one-third of sudden cardiac deaths (SCD) occurred in the first week (3-5 days) after MI and, in remaining two-thirds, 3 to 6 months after MI. Sensitivity, specificity, and positive predictive value of low EF, abnormal SAECG, abnormal HRV, and presence of NSVT in ambulatory electrocardiography were summarized in Table 2 and detailed below.

### Value of ejection fraction for postMI risk stratification

All the patients with arrhythmic events had ejection fraction of less than 40%. Sensitivity and specificity of EF<40% for predicting arrhythmic events was 100% and 54%, respectively (P<0.001).  Using ROC analysis, the probability of predicting SCD by an ideal observer based on EF<40% is 76.95% (chance level: 50%) 

### Value of SAECG for post MI risk stratification

Sensitivity and specificity of abnormal SAECG for predicting arrhythmic events is 67% and 75% respectively (P=0.0034). The probability of predicting SCD based on abnormal SAECG by an ideal observer is 70.83 (chance level: 50%).

### Value of HRV for post MI risk stratification

Abnormal HRV has sensitivity and specificity of 67% and 66% respectively for predicting SCD in post MI patients (P=0.025). Positive predictive value of abnormal HRV is 66.15 (chance level: 50%).

### Value of NSVT for post MI risk stratification

NSVT in this study was not significantly related to arrhythmic cardiac events (P=0.205).

### Effect of revascularization on incidence of MI-related sudden cardiac death

The probability of revascularization in patients with arrhythmic events and without arrhythmic events was 11% and 41%, respectively. These two probabilities are significantly different (P=0.036). The probability of SCD in patients who underwent revascularization was 2%, while in patients without revascularization it was 10%. Again these two chances are significantly different (P=0.03). Subgroup analysis showed that only CABGs was associated with significant reduction of fatal arrhythmias in post MI patients (P=0.029). In percutaneous coronary intervention (PCI) group the relationship between angioplasty and arrhythmia reduction was not significant (P=0.11).

### Effect of thrombolytics on incidence of MI-related sudden cardiac death

The chance of SCD in patients who received streptokinase was 4% and in those who did not receive streptokinase it was 8%. But this relationship was not statistically significant  (P=0.178).

## Discussion

In this study, EF<40% was the strongest predictor of arrhythmic events in postMI patients with sensitivity of 100%.  However, specificity of EF<40% is low (54%) and this limits ICD case selection based on low ejection fraction alone. The same result was obtained in several studies [9-14]. All of these studies have shown that left ventricular ejection fraction predicts outcome. Reduced left ventricular ejection fraction in most of these studies proved to be the most consistent independent risk factor for cardiac death in multivariate analyses.

SAECG and HRV are also significantly related to arrhythmic events but they seem to have lesser predicting power than EF<40%. For evaluating additive value of SAECG and HRV on predictive power of EF<40%, we designed a logistic model based on EF and then another model constructed based on EF plus SAECG and HRV. The two models were compared using likelihood ratio test and observed that the additional value of SAECG and HRV on EF<40% is marginally significant (P=0.036). Presence of late potentials and abnormal HRV have been correlated with the inducibility of VT and their prognostic value in post MI patients have also been established in previous studies [15-20]. However, results of recent studies leads to the SAECG having fallen out of favor recently [21].

In our study NSVT was not a predictor for post MI sudden death. This result is in contrast with result of the Munich and Berlin Infarction Study [9-22]. In the latter study, a Holter recording was performed in 1202 post MI patients before hospital discharge. Fourteen percent of patients with documented NSVT either died suddenly or developed symptomatic sustained VT or ventricular fibrillation (VF) within the next two years, compared with only 3.5% of patients without documentation of NSVT. The predictive value of rapid NSVT was even more impressive: if the rate of non-sustained ventricular tachycardia was ≥150 bpm (prevalence 3.7%), the rate of either sudden death or sustained ventricular tachyarrhythmias (VT or VF) was 22%, the relative risk being six times higher than the risk of patients without demonstration of rapid NSVT [23]. Therefore, lack of efficacy of NSVT for SCD prediction in our study may be explained by the definition used for NSVT in Holter and duration of recording.

The study showed significant reduction of arrhythmic events by revascularization. Further analysis showed that only CABGs was associated with significant reduction of fatal arrhythmias in post MI patients (P=0.029). In PCI group the relationship between angioplasty and arrhythmia reduction was not significant (P=0.11) although a trend toward reducing the risk was observed. Although TIMI-3 flow was achieved in all patients who had PCI, we think that this finding may be explained by relatively small number of patients (only 13%) and incomplete revascularization in PCI group. Again in patients received thrombolytic therapy in acute phase of MI, there was no significant decrease in arrhythmic cardiac events (P=0.178).

## Conclusions

Left ventricular EF less than 40% is the most important predictor of arrhythmic death among post MI patients. However, due to low specificity, other factors such as SAECG and HRV must be added to low EF for better risk stratification. NSVT was not significantly related to arrhythmic events in this study. Revascularization significantly reduces risk of sudden death in post MI patients. However only CABGs had meaningful effect on arrhythmic events and PCI failed to show this effect.

## Figures and Tables

**Table 1:**
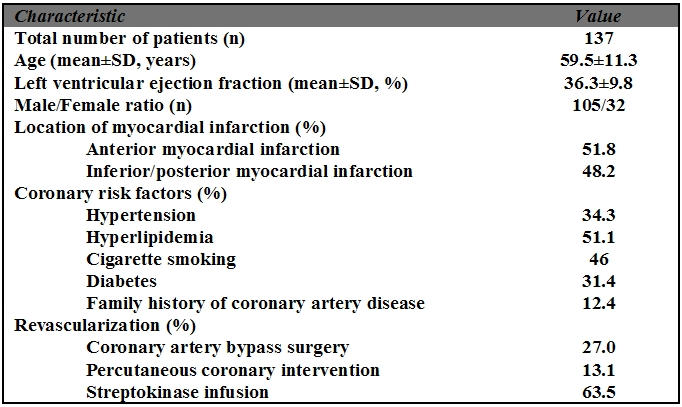
Characteristics of study population

**Table 2:**
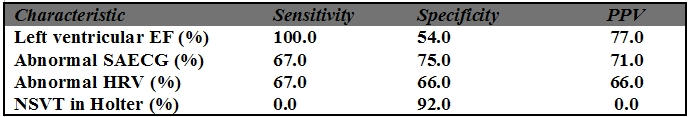
Sensitivity, specificity, and positive predictive value of myocardial infarction-related sudden cardiac death risk factors

Abbreviations: EF=ejection fraction; SAECG=signal averaged electrocardiography; HRV=heart rate variability; NSVT=nonsustained ventricular tachycardia
